# Multi-Chemical Profiling of Strawberry as a Traceability Tool to Investigate the Effect of Cultivar and Cultivation Conditions

**DOI:** 10.3390/foods9010096

**Published:** 2020-01-16

**Authors:** Raúl González-Domínguez, Ana Sayago, Ikram Akhatou, Ángeles Fernández-Recamales

**Affiliations:** 1 Department of Chemistry, Faculty of Experimental Sciences, University of Huelva, 21007 Huelva, Spain; ana.sayago@dqcm.uhu.es (A.S.); ikram.akhatou@alu.uhu.es (I.A.); 2 International Campus of Excellence CeiA3, University of Huelva, 21007 Huelva, Spain

**Keywords:** strawberry, traceability, sugars, organic acids, phenolic compounds, mineral elements, cultivar, cultivation system

## Abstract

The chemical composition of foods is tightly regulated by multiple genotypic and agronomic factors, which can thus serve as potential descriptors for traceability and authentication purposes. In the present work, we performed a multi-chemical characterization of strawberry fruits from five varieties (Aromas, Camarosa, Diamante, Medina, and Ventana) grown in two cultivation systems (open/closed soilless systems) during two consecutive campaigns with different climatic conditions (rainfall and temperature). For this purpose, we analyzed multiple components closely related to the sensory and health characteristics of strawberry, including sugars, organic acids, phenolic compounds, and essential and non-essential mineral elements, and various complementary statistical approaches were applied for selecting chemical descriptors of cultivar and agronomic conditions. Anthocyanins, phenolic acids, sucrose, and malic acid were found to be the most discriminant variables among cultivars, while climatic conditions and the cultivation system were behind changes in polyphenol contents. These results thus demonstrate the utility of combining multi-chemical profiling approaches with advanced chemometric tools in food traceability research.

## 1. Introduction

The composition of foods, in terms of nutrients, bioactive compounds, and other components, is tightly regulated by multiple factors, such as the genotype, geographical origin, environmental factors, and agronomic conditions. Therefore, this influences the sensory, nutritional, and nutraceutical properties of food products, which makes the implementation of quality control strategies mandatory to ensure their authenticity and traceability. In this vein, it should be noted that food quality and safety may be influenced by a myriad of factors throughout the entire supply chain, from initial food production to packaging, processing, and transport, until its final commercialization [1]. This is particularly important for processed foods, which usually require more complex operations and thus make the implementation of efficient traceability initiatives mandatory. To address these needs, novel and powerful analytical methods are requested by the food industry to accurately guarantee the authenticity and traceability of food products.

Strawberry (*Fragaria × ananassa* Duch.) is one of the most commonly consumed berry fruits around the world and is considered a functional food because of its chemical composition, which rich in essential and bioactive compounds. Strawberry has been demonstrated to lower post-prandial oxidative stress, hyperglycemia, hyperlipidemia, and inflammation, and its consumption has been associated with a reduced incidence of cardiovascular diseases (e.g., hypertension), cancer, and other diseases [2,3]. In this context, this berry fruit has been proposed as a potential ingredient for the production of nutraceutical products, such as beverages, flours, and powders [4]. The main soluble constituents of strawberry include sugars (e.g., glucose, fructose, and sucrose) and organic acids (e.g., citric and malic), which influence the final taste and flavor of this fruit [5]. Furthermore, strawberry is also a rich source of numerous bioactive compounds, such as dietary fibers, minerals, vitamins, and phenolic compounds [6,7]. In particular, polyphenols are known to elicit multiple biological activities, acting as natural antioxidants that protect the organism against free radicals [8]. Other pro-healthy compounds found in strawberries are vitamins and minerals, which intervene in a multitude of processes and chemical reactions inside the cells. For instance, potassium, a major element in strawberry, plays an important role in protection against cardiovascular diseases [9]. According to recent literature, this characteristic chemical profile of strawberry is largely influenced by multiple factors (e.g., cultivar, climate, and cultivation conditions) [7,10,11,12,13], evidencing its potential as a traceability tool.

In this work, we employed a multi-targeted profiling approach to characterize the chemical composition of strawberry, considering multiple compounds related to sensory and health characteristics of this berry fruit, including sugars, organic acids, polyphenols, and mineral elements. This multi-chemical profile was investigated as a potential tool for authentication and traceability purposes, with the aim of discriminating strawberry varieties grown under different climatic and agronomic conditions. For this purpose, complementary pattern recognition procedures were employed, including principal component analysis (PCA), linear discriminant analysis (LDA), soft independent model class analogy (SIMCA), and partial least squares discriminant analysis (PLS-DA).

## 2. Materials and Methods

### 2.1. Experimental Design and Sampling

Strawberry fruits (*Fragaria × ananassa* Duch.) were collected in two consecutive campaigns (years 2015 and 2016) from the same experimental plantations located in Huelva (southwest Spain), at the same commercial ripeness (>75% of the surface showing red color). The first campaign was characterized by higher total radiation, while in the second one, higher rainfall, and maximum and minimum temperatures were registered. Five varieties of strawberries, genetically characterized by the vendor (Aromas, Camarosa, Diamante, Medina, and Ventana) and grown in two soilless systems (closed and open systems, i.e., with and without recirculation of the nutrient solution, respectively), were investigated. Plants were grown in a polycarbonate-covered greenhouse using elevated horizontal troughs filled with coconut fiber as a substrate, and with natural daylight as a radiation source. The temperature ranged from 25 °C during the day to 8 °C at night, with relative humidity held at 75 ± 5%.

Several fruits (*n* = 10) were collected for each variety and cultivation system to generate a representative pooled sample. Immediately after harvesting, fruits were sorted, frozen in situ in a deep freezer, and shipped to the laboratory in polystyrene punnets. Then, fruits were washed, sepals were dissected, and pooled fruits (*n* = 10) were gently homogenized by using a kitchen mixer to obtain a puree (approximately 100–150 mL). Samples were subsequently aliquoted and stored for up to 2 months at −21 °C, until further analysis. For each study condition (i.e., cultivar, campaign, and cultivation conditions), three replicates (i.e., three pooled and homogenized samples) were prepared.

### 2.2. Analysis of Sugars and Organic Acids

Sugars and organic acids were analyzed using an Agilent 110 series high-performance liquid chromatography (HPLC) system coupled to ultraviolet (UV) and refractive index (RI) detectors (Agilent Technologies, Santa Clara, CA, USA), following the methodology previously described [7]. Approximately 1 g of the homogenate was accurately weighed, diluted to 10 mL with ultrapure water (Millipore, Bedford, Massachusetts, MA, USA), and centrifuged at 10,000 rpm for 10 min (BHG-Hermle Z 365, Wehingen, Germany). The supernatant was filtered through a 0.45 μm PVDF (polyvinylidene difluoride) filter prior to HPLC analysis.

In a single chromatographic run, three sugars (glucose, fructose, and sucrose) and six organic acids (oxalic, citric, tartaric, malic, succinic, and lactic) were separated using a Metacarb 87H hydrogen-form cation-exchange resin-based column (300 × 7.8 mm internal diameter, i.d.) packed with sulfonated polystyrene. A total of 5 mM of sulfuric acid was delivered in isocratic mode at a 0.5 mL min^−1^ flow rate for 15 min, and the injection volume was 20 μL. UV detection of organic acids was performed at 210 nm, while sugars were analyzed by using the RI detector. Identifications were accomplished by comparing retention times (and UV spectra for organic acids) with those of reference standards.

### 2.3. Analysis of Phenolic Compounds

Homogenized fruits (5.0 g) were dissolved with 25 mL of methanol, sonicated for 30 min, and then centrifuged at 10,000 rpm for 10 min at 4 °C. Supernatants were concentrated by using a rotary evaporator at 40 °C, and the residues were re-dissolved in 3 mL of 50% methanol (*v*/*v*). The concentrated extracts were filtered through 0.45 μm PVDF filters, and 20 μL was injected into a reverse phase Ultrabase C18 column (2.5 μm, 100 mm × 4.6 mm i.d.), following the methodology described elsewhere [10]. For the analysis of colorless flavonoids and phenolic acids, elution solvents were water:methanol:acetic acid (93:5:2, *v*/*v*/*v*) (eluent A) and methanol:acetic acid (98:2, *v*/*v*) (eluent B), which were delivered as follows: 0–29 min, 40% B; 29–34.8 min, 40–60% B; 34.8–37.7 min, 60–75% B; 37.7–40.6 min, 75–100% B; 40.6–46.4 min, 0% B. For anthocyanins, the mobile phase consisted of 10% (*v*/*v*) aqueous formic acid (eluent A) and methanol (eluent B), using the following gradient program: 0–0.70 min, 5% B; 0.70–16.60 min, 5–50% B; 16.60–18.60 min, 50–95% B; 18.60–20.60 min, 95% B. The flow rate was 0.8 mL min^−1^, and the column temperature was set at 20 and 30 °C for non-anthocyanin and anthocyanin compounds, respectively.

The identification of phenolic compounds was achieved by comparing their retention times and UV spectra with those for commercial standards. For quantification, the following wavelengths were employed: 260 nm for ellagic acid and derivatives, 280 nm for benzoic acids and flavan-3-ols, 320 nm for cinnamic acids, 360 nm for flavonols, and 520 nm for anthocyanins.

### 2.4. Analysis of Mineral Elements

For mineral content analysis, 0.5 g of fruit was placed in a Teflon vessel and digested with 3 mL of a mixture of nitric and hydrochloric acids, both 1.5 M. Digestion was carried out for 2 min using a microwave furnace at 250 W. After cooling, the digest was filtered, transferred to a 25 mL flask, and made-up with ultrapure water.

The major (i.e., Ca, Mg, K, P, and S) and trace (i.e., Al, As, Cd, Cr, Cu, Fe, Hg, Ni, Pb, Ba, Mn, Na, Sr, V, and Zn) minerals were determined by inductively coupled plasma optical emission spectrometry (ICP-OES) using a Jobin-Yvon Ultima 2 ICP spectrometer with an ultrasonic nebulizer (U6000 AT+, Cetac). The instrument was operated at the following conditions: radio frequency, 27 MHz; operating power, 1200 W; plasma argon flow rate, 2 L min^−1^; auxiliary gas flow rate, 2 L min^−1^; nebulizer gas flow rate, 0.02 L min^−1^; nebulizer pressure, 1 bar; rinsing time, 35 s; rinsing pump speed, high; transfer time, 60 s; stabilization time, 20 s; and transfer pump speed, high. ICP Multi Element Standard IV and VI CertiPur^®^ (Merck) were used to prepare reference solutions.

### 2.5. Statistical Analysis

One-way analysis of variance (ANOVA), multivariate analysis of variance (MANOVA), and pattern recognition techniques, including principal component analysis (PCA), linear discriminant analysis (LDA), soft independent modeling of class analogy (SIMCA), and partial least squares discriminant analysis (PLS-DA), were carried out to investigate the differences among strawberry varieties and/or cultivation systems. All statistical analyses were conducted on Statistica 7.1 (StatSoft Inc., Tulsa, Oklahoma, OK, USA) and SIMCA-P™ 11.5 (UMetrics AB, Umeå, Sweden).

## 3. Results and Discussion

### 3.1. Multi-Chemical Profiling of Strawberry

Mean concentrations for all the analyzed compounds (i.e., sugars, organic acids, polyphenols, and mineral elements) are listed in Table 1 for the five strawberry cultivars investigated. Soluble sugars identified and quantified in strawberry fruits were fructose, glucose, and sucrose; monosaccharides were the major species in all varieties, except for “Camarosa”, which showed higher sucrose contents. The ratio of fructose to glucose content was about the same, regardless of the cultivar, in agreement with our previous study findings [10]. With regards to organic acids, citric acid was the most concentrated metabolite, followed by malic acid, in consonance with previous studies [7,11]. In agreement with results found in the literature, anthocyanins were the predominant polyphenol class in strawberry [14], followed by phenolic acids, with pelargonidin 3-glucoside, pelargonidin 3-rutinoside, and cyanidin 3-glucoside being the three major anthocyanin species [8,15], which were found at similar levels to those reported by Crespo et al. [16]. The mineral profile was mainly dominated by five major elements—K, P, Ca, Na, and Mg—with potassium showing the highest concentrations (average content of 2834.5 mg kg^−1^). Phosphorous, calcium magnesium, and sodium were also present in high concentrations, representing approximately 20% of the total mineral content, while other elements (Fe, Cu, Zn, and Sr) accounted for less than 1% of the mineral profile. It should be noted that these results are in line with previous findings [7].

Multivariate analysis of variance (MANOVA) was applied to test the effects of the cultivar and cultivation system on the chemical profile, and analysis of variance (ANOVA) with a Tukey HSD post hoc test was used to evaluate the statistical significance of the differences for each compound or element measured. The multivariate test showed that both factors have a significant effect on the content of sugars, organic acids, and polyphenols (*p* < 0.001), but not on the mineral profile (*p* > 0.1 and *p* > 0.5 for the variety and cultivation system, respectively). Univariate results for each variable are shown in Table 1. “Camarosa” and “Ventana” were found to be the richest cultivars in total sugars and organic acids. In particular, “Camarosa” strawberries showed the highest content of sucrose and malic acid. The “Ventana” cultivar presented the richest profile in phenolic acids, mainly dominated by ellagic acid, while “Camarosa” and “Aromas” varieties showed higher concentrations of total polyphenols, mainly anthocyanins.

### 3.2. Application of Pattern Recognition Tools for Selecting Chemical Descriptors of Cultivar and Agronomic Conditions

Several chemometric techniques, including unsupervised and supervised pattern recognition procedures, were employed to achieve a reliable differentiation between strawberry samples according to the cultivar, cultivation system, and/or campaign.

**Figure 1:**
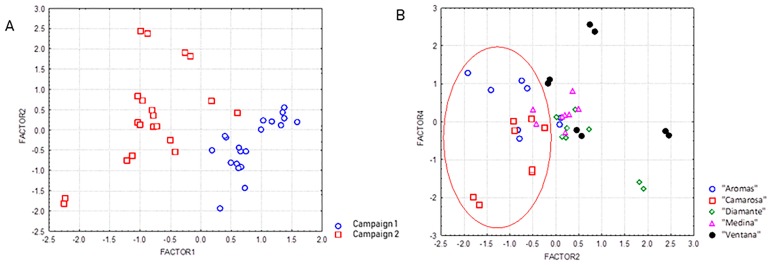
Principal component analysis (PCA) score plots showing the projection of strawberry samples in the plane defined by the following principal components: (**A**) PC1 vs. PC2, separation of samples according to the campaign; (**B**) PC2 vs. PC4, separation of samples according to the cultivar.

A preliminary data exploration was carried out by principal component analysis (PCA), using autoscaled data and only considering the principal components (PCs) with eigenvalues greater than 1. This PCA model allowed 84% of the total variance to be explained with five components. As shown in the scores plot built using the two first principal components (Figure 1A), a clear separation was observed along the PC1 among samples collected in the two consecutive campaigns. The first PC explained 28% of the variance, and was positively related to fructose and tartaric acid, and negatively associated with pelargonidin 3-glucoside, total flavonoids, and total polyphenols. That is, the content of anthocyanins and total polyphenols was greater during the second campaign, when rainfall, and maximum and minimum temperatures were higher, whereas fructose and tartaric acid contents were more abundant in the first campaign, when total radiation was higher. In this vein, it has previously been described that the content of many phenolic compounds and the antioxidant capacity increase in berry fruits as the temperature increases [17]. Moreover, a low light intensity and high temperatures have also been demonstrated to provoke a decreased synthesis of sugars and ascorbic acid [7,11,18]. On the other hand, the plotting of the second and fourth PCs provided a certain differentiation, depending on the cultivar (Figure 1B), with “Camarosa” and “Aromas” varieties distributed on the left side of the projection, and the rest of the samples located on the right side. The most relevant compounds contributing to this separation were anthocyanins (increased in “Camarosa” and “Aromas”) and phenolic acids (decreased in “Camarosa” and “Aromas)”, in accordance with the results obtained by ANOVA.

After this preliminary data exploration, several supervised chemometric tools were employed to build classification models with the aim of assessing the potential of the multi-chemical profile investigated in this work to authenticate strawberries according to the variety and cultivation conditions. For this purpose, multiple supervised pattern recognition procedures have recently been proposed in food research to solve authentication problems for various foods with a high commercial value, such as strawberry [11,15], olive oil [19,20,21], or wine [22,23]. In the present study, three complementary statistical techniques were tested: linear discriminant analysis (LDA), soft independent modeling of class analogy (SIMCA), and partial least squares discriminant analysis (PLS-DA).

**Figure 2:**
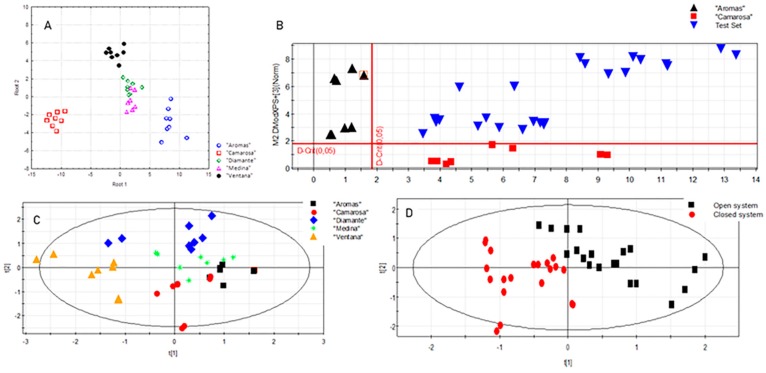
Results obtained from supervised chemometric modeling. (**A**) Linear discriminant analysis (LDA) scores plot showing the distribution of samples in the plane defined by the two first principal components using the cultivar as the categorical variable; (**B**) Soft independent model class analogy (SIMCA) Coomans plots for the classification of strawberry samples according to the cultivar: “Aromas” vs. ”Camarosa”; (**C**) Partial least squares discriminant analysis (PLS-DA) scores plot showing the distribution of samples in the plane defined by the two first principal components using the cultivar as the categorical variable; (**D**) PLS-DA scores plot showing the distribution of samples in the plane defined by the two first principal components using the cultivation system as the categorical variable.

Linear discriminant analysis (LDA) was first applied to all the study variables, yielding a model capable of explaining 96% of the total variance with a 95% prediction ability. Applying forward stepwise analysis, cyanidin 3-glucoside, pelargonidin 3-rutinoside, p-coumaric acid, phosphorous, malic acid, caffeic acid, and quercetin were identified as the most discriminant variables among cultivars. As shown in Figure 2A, all samples were correctly classified, with the exception of two samples of “Medina” cultivar, which were classified as “Diamante”. In line with the results from PCA, “Aromas” and “Camarosa” cultivars were clearly differentiated from the rest of the samples along the first root, while the second one described almost complete separation between the other three cultivars.

Soft independent modeling of class analogy (SIMCA) was subsequently applied to the same data matrix used in LDA, with the aim of looking for possible overlap among the study groups. Using a seven-fold cross-validation procedure, 3-PC-based models were obtained explaining 96.4%, 94.5%, 95.0%, 92.5%, and 97.2% of variance for the classes “Aromas”, “Camarosa”, “Diamante”, “Medina”, and “Ventana”, respectively. These models also provided very good results in terms of their prediction ability, with 86.7%, 81.5%, 80.8%, 71.3%, and 88.5% correct prediction for the five cultivars. In this line, representation of the corresponding Coomans plot showed a correct classification of strawberries according to the variety based on their chemical composition (Figure 2B). However, SIMCA modeling did not provide suitable results for the classification of strawberry samples according to agronomic conditions, with samples appearing in the overlapping area from the Coomans plots (figure not shown).

Finally, partial least squares discriminant analysis (PLS-DA) was also employed as a more powerful technique for class differentiation and for the selection of the most discriminant variables. A five-component model was obtained with a good quality of fit (*R*^2^_X_ = 0.744) and predictive ability (Q^2^ = 0.413) for the classification of strawberry samples according to the cultivar (Figure 2C). The most important chemical descriptors driving this separation were anthocyanins and phenolic acids, in line with previous findings from ANOVA and LDA. Interestingly, PLS-DA modeling also enabled the discrimination of samples grown in the two cultivation systems (i.e., open and closed soilless systems). The PLS-DA model explained 70.2% of the variance (Figure 2D), with p-hydroxybenzoic acid, ferulic acid, unknown derivatives of pelargonidin, glucose, pelargonidin acetylglucoside, and cyanidin 3-glucoside being the most discriminant variables.

## 4. Conclusions

In this work, we have evaluated the potential of combining multi-chemical profiling and complementary statistical techniques to investigate the effect of the genotype and cultivation conditions on the chemical composition of strawberry fruits. The five cultivars investigated showed clear differences in the content of anthocyanins, phenolic acids, sucrose, and malic acid. On the other hand, climatic conditions (e.g., rainfall and temperature) were responsible for slight changes in the polyphenolic profile, with an increased content of anthocyanins and total polyphenols in strawberry fruits grown under higher rainfall and more extreme temperatures. Similarly, the cultivation conditions (i.e., open/closed soilless system) also induced minor changes in concentrations of several anthocyanins and phenolic acids. The present work therefore demonstrates that multi-chemical profiling can be used to differentiate among strawberry cultivars grown under different agronomic conditions, thus showing a great applicability for food traceability. In future studies, this approach could also be tested to search for characteristic patterns associated with the geographical origin, ripeness status, and other factors related to food production.

## Figures and Tables

**Table 1:** Concentrations (expressed as the mean ± standard deviation) of sugars (g kg^−1^), organic acids (g kg^−1^), phenolic compounds (mg kg^−1^), and mineral elements (mg kg^−1^) in each strawberry cultivar, and *p* values obtained by ANOVA.

Compounds	Aromas	Camarosa	Diamante	Medina	Ventana	*p* Value
sucrose	6.9 ± 4.8	14.1 ± 2.3	9.2 ± 1.7	6.7 ± 3.4	10.0 ± 3.1	0.0003
glucose	11.9 ± 4.4	11.7 ± 3.2	12.4 ± 3.0	12.3 ± 3.9	14.6 ± 3.9	0.4836
fructose	11.6 ± 4.1	11.0 ± 2.8	11.5 ± 2.4	11.4 ± 3.8	13.3 ± 3.4	0.6800
**Total Sugars**	**30.5 ± 12.8**	**36.7 ± 6.3**	**33.1 ± 5.7**	**30.4 ± 10.2**	**37.9 ± 8.7**	**0.3383**
ascorbic acid	0.1 ± 0.04	0.2 ± 0.1	0.2 ± 0.02	0.2 ± 0.08	0.2 ± 0.1	0.4145
citric acid	5.1 ± 2.0	6.3 ± 0.8	5.3 ± 1.1	4.7 ± 1.4	5.3 ± 0.8	0.1937
tartaric acid	0.08 ± 0.08	0.1 ± 0.04	0.2 ± 0.06	0.07 ± 0.09	0.2 ± 0.07	0.0959
malic acid	0.5 ± 0.1	2.4 ± 0.4	0.6 ± 0.2	0.5 ± 0.1	0.7 ± 0.2	0.0871
**Total Acids**	**5.8 ± 2.2**	**8.9 ± 2.9**	**6.2 ± 1.3**	**5.5 ± 1.5**	**6.4 ± 0.8**	**0.0074**
pelargonidin derivative 1	0.9 ± 0.3	0.8 ± 0.2	0.6 ± 0.2	0.7 ± 0.3	0.7 ± 0.4	0.0750
cyanidin 3-glucoside	6.4 ± 1.6	4.0 ± 0.8	3.0 ± 1.1	3.8 ± 0.2	1.4 ± 0.6	0.0000
pelargonidin 3-glucoside	120.9 ± 17.7	117.2 ± 29.9	72.4 ± 3.3	102.7 ± 30.9	86.1 ± 22.2	0.0003
pelargonidin 3-rutinoside	7.4 ± 1.6	15.8 ± 5.1	5.2 ± 1.0	6.2 ± 0.7	6.7 ± 2.5	0.0000
pelargonidin derivative 2	0.7 ± 0.2	0.6 ± 0.3	0.6 ± 0.2	0.7 ± 0.09	0.8 ± 0.4	0.8180
pelargonidin acetate	3.0 ± 0.4	2.3 ± 0.7	1.4 ± 0.2	2.1 ± 0.8	1.1 ± 0.4	0.0000
**Total Anthocyanins**	**139.4 ± 18.3**	**140.9 ± 35.9**	**83.3 ± 1.9**	**116.2 ± 32.3**	**96.7 ± 25.1**	**0.0001**
p-hydroxybenzoic acid	0.6 ± 0.1	1.4 ± 0.3	0.8 ± 0.9	0.3 ± 0.02	0.5 ± 0.03	0.0139
caffeic acid	0.4 ± 0.1	0.6 ± 0.2	0.2 ± 0.01	0.5 ± 0.1	0.9 ± 0.2	0.0001
p-coumaric acid	7.8 ± 1.7	6.6 ± 3.1	4.2 ± 2.2	5.8 ± 1.3	19.3 ± 6.1	0.0000
ferulic acid	0.08 ± 0.02	0.2 ± 0.07	0.2 ± 0.02	0.1 ± 0.04	0.4 ± 0.08	0.0078
ellagic acid	39.3 ± 10.3	35.8 ± 10.5	54.3 ± 23.8	45.8 ± 22.3	63.4 ± 25.9	0.1295
**Total Phenolic Acids**	**48.1 ± 11.1**	**44.6 ± 13.5**	**59.8 ± 24.5**	**52.6 ± 22.4**	**84.4 ± 36.3**	**0.0129**
quercetin	1.4 ± 0.08	1.5 ± 0.2	0.9 ± 0.2	0.9 ± 0.3	0.7 ± 0.1	0.0249
Kaempferol O-glucoside	23.0 ± 7.2	29.5 ± 10.3	18.3 ± 6.0	21.2 ± 9.5	30.3 ± 8.3	0.0262
**Total Flavonols**	**24.4 ± 7.2**	**31.0 ± 10.4**	**19.3 ± 6.1**	**22.2 ± 9.9**	**31.0 ± 7.9**	**0.0237**
P	224.3 ± 35.6	251.6 ± 11.3	196.2 ± 29.5	219.2 ± 21.8	218.5 ± 16.4	0.0025
Ba	0.6 ± 0.2	0.5 ± 0.06	0.4 ± 0.06	0.4 ± 0.02	0.4 ± 0.04	0.9586
Ca	210.7 ± 24.1	244.2 ± 35.4	156.4 ± 20.1	195.4 ± 33.2	235.2 ± 28.6	0.9732
Cr	0.1 ± 0.02	0.06 ± 0.01	0.06 ± 0.02	0.05 ± 0.01	0.2 ± 0.03	0.9932
Cu	4.6 ± 1.9	4.8 ± 1.1	4.8 ± 1.4	4.9 ± 1.4	5.4 ± 1.3	0.9915
Fe	7.8 ± 1.2	7.6 ± 1.1	5.8 ± 1.6	8.7 ± 1.1	7.5 ± 1.9	0.9723
K	2843.5 ± 287.6	2788.2 ± 357.4	2098.1 ± 210.2	2844.8 ± 351.9	3597.9 ± 443.5	0.9263
Mg	226.7 ± 26.9	179.3 ± 26.0	142.5 ± 20.1	167.5 ± 23.7	222.6 ± 30.7	0.9526
Mn	8.8 ± 1.9	6.9 ± 1.0	5.9 ± 1.9	6.8 ± 1.1	9.9 ± 1.1	0.9394
Na	189.1 ± 28.9	116.0 ± 23.7	98.3 ± 21.5	88.9 ± 17.4	126.2 ± 16.1	0.9000
Ni	0.3 ± 0.06	0.3 ± 0.02	0.3 ± 0.07	0.3 ± 0.03	0.3 ± 0.05	0.9890
Sr	6.0 ± 1.0	3.6 ± 1.7	3.1 ± 1.5	4.8 ± 1.8	5.6 ± 1.7	0.8802
Zn	3.2 ± 0.9	7.5 ± 0.8	3.74 ± 0.49	3.47 ± 0.33	4.26 ± 0.30	0.5551

ANOVA, One-way analysis of variance.
